# Endoplasmic reticulum retention and degradation of a mutation in *SLC6A1* associated with epilepsy and autism

**DOI:** 10.1186/s13041-020-00612-6

**Published:** 2020-05-12

**Authors:** Jie Wang, Sarah Poliquin, Felicia Mermer, Jaclyn Eissman, Eric Delpire, Juexin Wang, Wangzhen Shen, Kefu Cai, Bing-Mei Li, Zong-Yan Li, Dong Xu, Gerald Nwosu, Carson Flamm, Wei-Ping Liao, Yi-Wu Shi, Jing-Qiong Kang

**Affiliations:** 1 Institute of Neuroscience and Department of Neurology of the Second Affiliated Hospital of Guangzhou Medical University; Key Laboratory of Neurogenetics and Channelopathies of Guangdong Province and the Ministry of Education of China, Guangzhou, 510260 China; 2 The Neuroscience Program, Vanderbilt University Medical Center, Nashville, TN 37232 USA; 3 Department of Anesthesiology, Vanderbilt University Department of Anesthesiology, Vanderbilt University, Nashville, TN 37232 USA; 4 Department of Electrical Engineering & Computer Science and Christopher S. Bond Life Sciences Center, University of Missouri, Columbia, MO 65211 USA; 5 Department of Neurology, Vanderbilt University Medical Center, Nashville, USA; 6 Department of Neurology, Affiliated Hospital, Nantong University, Nantong, 226001 Jiangsu China; 7 Neuroscience Graduate Program, Vanderbilt-Meharry Alliance, Vanderbilt University, Nashville, TN 37235 USA; 8 Department of Pharmacology, Vanderbilt University, Vanderbilt Kennedy Center of Human Development, Vanderbilt Brain Institute, 6147 MRBIII, 465 21st Ave. South, Nashville, TN 37232 USA

**Keywords:** Endoplasmic reticulum, Degradation, Mutation, GABA transporter 1, Protein stability, ^3^H GABA uptake, Autism, Epilepsy

## Abstract

Mutations in *SLC6A1*, encoding γ-aminobutyric acid (GABA) transporter 1 (GAT-1), have been recently associated with a spectrum of epilepsy syndromes, intellectual disability and autism in clinic. However, the pathophysiology of the gene mutations is far from clear. Here we report a novel *SLC6A1* missense mutation in a patient with epilepsy and autism spectrum disorder and characterized the molecular defects of the mutant GAT-1, from transporter protein trafficking to GABA uptake function in heterologous cells and neurons. The heterozygous missense mutation (c1081C to A (P361T)) in *SLC6A1* was identified by exome sequencing. We have thoroughly characterized the molecular pathophysiology underlying the clinical phenotypes. We performed EEG recordings and autism diagnostic interview. The patient had neurodevelopmental delay, absence epilepsy, generalized epilepsy, and 2.5–3 Hz generalized spike and slow waves on EEG recordings. The impact of the mutation on GAT-1 function and trafficking was evaluated by ^3^H GABA uptake, structural simulation with machine learning tools, live cell confocal microscopy and protein expression in mouse neurons and nonneuronal cells. We demonstrated that the GAT-1(P361T) mutation destabilizes the global protein conformation and reduces total protein expression. The mutant transporter protein was localized intracellularly inside the endoplasmic reticulum (ER) with a pattern of expression very similar to the cells treated with tunicamycin, an ER stress inducer. Radioactive ^3^H-labeled GABA uptake assay indicated the mutation reduced the function of the mutant GAT-1(P361T), to a level that is similar to the cells treated with GAT-1 inhibitors. In summary, this mutation destabilizes the mutant transporter protein, which results in retention of the mutant protein inside cells and reduction of total transporter expression, likely via excessive endoplasmic reticulum associated degradation. This thus likely causes reduced functional transporter number on the cell surface, which then could cause the observed reduced GABA uptake function. Consequently, malfunctioning GABA signaling may cause altered neurodevelopment and neurotransmission, such as enhanced tonic inhibition and altered cell proliferation in vivo. The pathophysiology due to severely impaired GAT-1 function may give rise to a wide spectrum of neurodevelopmental phenotypes including autism and epilepsy.

## Introduction

Autism or autism spectrum disorder (ASD) is a common childhood-onset neurodevelopmental condition with a strong genetic basis. The genetic architecture of ASD consists of rare de novo or inherited variants in hundreds of genes and common polygenic risks at thousands of loci. Genetic advances indicate ASD susceptibility genes are enriched for roles in early brain development and in cortical cell types [17], as well as in synaptic formation and function [13]. Importantly, ASD has a high comorbidity with epilepsy, suggesting common genetic and molecular susceptibility underlying both epilepsy and ASD [22]. This comorbidity also suggests findings from epilepsy may provide unique insights into understanding ASD.

The GABAergic pathway is likely a converging pathway for many gene mutations associated with ASD. This concept is rooted in the fact that multiple epilepsy syndromes are comorbid with ASD or autistic features [13, 15]. *SLC6A1*, encoding γ-aminobutyric acid (GABA) transporter 1 (GAT-1), is one such gene commonly associated with epilepsy and ASD. This is not surprising because GAT-1 is one of the major GABA transporters in the brain and a key component of GABA signaling. Impaired GAT-1 function may result in altered GABA levels and the excitation-inhibition imbalance that is a hallmark for autism [20, 33]. GABA is a neurotrophic signal that is critical for early brain development, including regulation of neural stem cell proliferation [3, 4]. It is plausible that impaired GABA signaling due to mutations in GABA_A_ receptor genes or GAT-1 can affect the fundamental properties of the progenitor cells such as proliferation and differentiation. In epilepsy, impaired GABAergic signaling is a converging pathway of pathophysiology for epilepsy genes, including both ion channel and non-ion channel genes [21]. GAT-1 is a major GABA transporter subtype of sodium- and chloride-dependent transporters and is localized in GABAergic axons and nerve terminals. Unlike GABA_A_ receptors that directly conduct postsynaptic GABAergic currents, GAT-1 influences GABAergic synaptic transmission by clearance and re-uptake of GABA from the synapse [14].

Since the first report of *SLC6A1* mutations in myoclonic atonic epilepsy (MAE), several studies have identified a number of mutations in *SLC6A1* associated with two prominent features: intellectual disability (ID) and a wide spectrum of epilepsy [9, 19]. A recent study also reported a *SLC6A1* mutation causes a milder phenotype, characterized by a learning disorder without ID, nonspecific dysmorphisms, and an electroencephalogram (EEG) picture closely resembling that of myoclonic-atonic epilepsy with brief absence seizures later on [38]. We previously reported *SLC6A1(G234S)* associated with Lennox-Gastaut syndrome (LGS) [8]. Because LGS is often associated with mutations in *GABRB3*, it is intriguing to find *SLC6A1* also associated with LGS. Overlapping clinical and molecular phenotypes of mutations in *SLC6A1* and *GABRB3* are further suggested by our previous study that a signal peptide variation in *GABRB3* is associated with ASD with maternal transmission in multiple Caucasian families [13]. However, this area merits further elucidation.

In this study, we evaluated the impact of a novel mutation (P361T) associated with epilepsy and ASD by characterizing the mutant protein trafficking and function in different cell types including mouse neurons. Additionally, we thoroughly evaluated patient disease history, seizure phenotype, EEG, and ASD phenotype. We compared the wildtype and mutant transporter with protein structure modeling via machine learning based prediction, ^3^H radioactive GABA uptake assay, and protein expression and subcellular localizations via confocal microscopy, in both heterologous cells and mouse cortical neurons. This study provides molecular mechanisms underlying how a defective GAT-1 can cause ASD in addition to epilepsy and expands our knowledge for understanding the pathophysiology underlying the comorbidity of ASD and epilepsy.

## Methods

### Patient with autism and epilepsy

The patient and her unaffected family members were first recruited at the Epilepsy Center and then evaluated in the clinical psychology clinic of the Second Affiliated Hospital of Guangzhou Medical University. The collected clinical data included age of onset, a detailed developmental history, autistic behaviors, seizure types and frequency, response to antiepileptic drugs (AEDs), family history, and general and neurological examination results. Brain magnetic resonance imaging (MRI) scans were performed to exclude brain structure abnormalities. Video electroencephalography (EEG) was examined repeatedly and the results were reviewed by two qualified electroencephalographers.

Autistic features were assessed and diagnosed by psychologists using Autism Diagnostic Interview Revised (ADI-R) [51] and Autism Diagnostic Observation Schedule-Genetic (ADOS-G) [30]. Individuals with the scores of ADI-R and ADOS greater than their corresponding threshold scores of ASD (cut-off) are considered to have ASD. To assess different aspects of the behaviors, developmental skills, and neuropsychological development of the patient, the third edition of Chinese Psychoeducational Profile (CPEP-3) (a modified version of Psychoeducational Profile – Revised (PEP-3)) [48, 49] and the Gesell Developmental Schedule were performed by the same psychologists. ASD was diagnosed according to the fifth edition of the *Diagnostic and Statistical Manual of Mental Disorders* (DSM-5), and the tenth edition of the *International Classification of Diseases* (ICD-10). When a patient meets DSM-5 and ICD-10 criteria for deficits in all three areas—communication, social interaction, and repetitive behaviors—a diagnosis of ASD is made. Epileptic seizures and epilepsy syndromes were diagnosed and classified according to the criteria of the Commission on Classification and Terminology of the International League Against Epilepsy (1989, 2001, and 2010).

This study was approved by the ethics committee of the Second Affiliated Hospital of Guangzhou Medical University, and written informed consent was obtained from the parents.

### Genetic data analysis

Blood samples of the patient, her parents, and her brother were collected. Genomic DNA was extracted from the peripheral blood using the Qiagen Flexi Gene DNA Kit (Qiagen, Germany). The SureSelect Human All Exon 50 Mb kit (Agilent Technologies Santa Clare, CA) was used to capture the exon regions of the genome. The DNA samples were sequenced using Illumina Hiseq 2000 sequencing system with 90-base pair reads, and the massively parallel sequencing was performed with more than 125 times average depth and more than 98% coverage of the target region.

The raw data were aligned to the human reference genome (GRCh37) using SOAP aligner (https://github.com/Shujia Huang/SOAPaligner). To obtain the whole list of potential pathogenic variants, a step-by-step filtering was conducted [41]. In brief, 1) Population-based filtration retained variants with a minor allele frequency (MAF) < 0.005 in the 1000 Genomes Project, Exome Aggregation Consortium (ExAC), ExAC-East Asian Population (ExAC-EAS), and Genome Aggregation Database (gnomAD). 2) Functional prediction-based filtration retained missense, nonsense, indel, frameshift, and splice variants based on computational and predictive data. Potential pathogenic variants were flagged if predicted as damaging by SIFT (http://sift.jcvi.org/), PolyPhen2 (http://genetics.bwh.harvard.edu/pph2/), and Mutation Taster (http:// mutationtaster.org/). 3) Inheritance-based filtration retained variants consistent with inherited models.

PCR-Sanger sequencing was performed to validate the candidate variant on ABI 3730 sequence (Applied Biosystems, Foster City, CA, USA). The primers were 5′-CCTCACTGGTCCTCTTGC-3′ and 5′-CTGTTTACTCGGGGACTG-3′. A total of 296 healthy volunteers were recruited as normal controls.

### The cDNAs for coding GABA transporter 1

The plasmid cDNA encoding enhanced yellow fluorescent protein (EYFP)-tagged rat GAT-1 was sub-cloned into the expression vector pcDNA3.1(+). Replications of patient GAT-1 mutations were cloned via a standard molecular cloning process. QuikChange Site-directed Mutagenesis kit was utilized to introduce the GAT-1(P361T) mutation into wildtype GAT-1 proteins. The product was then amplified via polymerase chain reaction and transformed using DHα competent cells and finally plated. A clone was chosen and grown overnight, replicating the cDNA. The GAT-1(P361T) mutation was confirmed by DNA sequencing. Both the wildtype and the mutant cDNAs were prepared with Qiagen Maxiprep kit.

### Polyethylenimine (PEI) transfection

Standard transfection protocols were performed using human embryonic kidney 293 T (HEK293T) cells [8]. 24 h before transfection HEK293T cells were split equally into plates. During transfections, 1 μg of the cDNAs was used and combined with Dulbecco modified Eagle medium (DMEM) and a PEI/DMEM mixture. Transfected HEK293T cells incubated for 48 h. After incubation, proteins were harvested as described below.

### Western blot analysis of total GAT-1 protein

Briefly, HEK293T cells were seeded in 60 mm^2^ dishes 1 day before transfection to avoid cell detachment. Live, transfected cells were washed with phosphate buffered saline (1 ***×*** PBS, pH 7.4) 3 times and then cells were lysed in RIPA buffer (20 mM Tris, 20 mM EGTA, 1 mM DTT, 1 mM benzamidine), supplemented with 0.01 mM PMSF, 0.005 μg/mL leupeptin, and 0.005 μg/mL pepstatin for 30 min at 4 °C. The samples were then subject to protein concentration determination and followed by SDS-PAGE. Membranes were incubated with primary rabbit polyclonal antibodies against GAT-1 (Alomone Labs, AGT-001 or Synaptic System, 274,102 at 1:200 dilution).

### Neuronal cultures and transfection in neurons

Mouse cortical neuronal cultures and transfection were prepared as previously described [24, 26]. Mouse neurons were cultured from postnatal day 0 mouse pups. The neurons were plated at a density of 2 × 10^5^ for western blot in plating media that contained 420 mL DMEM, 40 mL F12, 40 mL fetal bovine serum, 1 mL penicillin and streptomycin, and 0.2 mL L-Glutamine (200 mM) for 4 h. Neurons were then maintained in Neurobasal media that contained B27 supplement (50:1), L-Glutamine (200 mM), and 1 mL penicillin and streptomycin. Neurons were transfected with 15 μg cDNA at day 5–7 in culture with calcium phosphate and were harvested 8–10 days after transfection. Four 100 mm^2^ dishes of neurons were transfected with either the wildtype or the mutant GAT-1^YFP^ cDNAs in each experiment to ensure enough proteins for immunoblotting assay due to low transfection efficiency in neurons.

### Radioactive ^3^H-labeled GABA uptake assay

The radioactive ^3^H-labeled GABA uptake assay in HEK293T and HeLa cells was modified from previous studies [8, 28]. Briefly, cells were cultured in 5 mm^2^ dishes 3 days before the GABA uptake experiment in DMEM with 10% fetal bovine serum and 1% penicillin/streptomycin. The cells were then transfected with equal amounts of the wildtype or the mutant GAT-1(P361T) cDNAs (1 μg) for each condition at 24 h after plating. GABA uptake assay was carried out 48 h after transfection. The cells were incubated with preincubation solution for 15 min and then incubated with preincubation solution containing 1μci/ml ^3^H GABA and 10 μM unlabeled GABA for 30 min at room temperature. After washing, the cells were lysed with 0.25 N NaOH for 1 h. Acetic acid glacial was added and lysates were then determined on a liquid scintillator with QuantaSmart. The flux of GABA (pmol/μg/min) was averaged with at least triplets for each condition at each transfection. The average counting was taken as *n* = 1. The untransfected condition was taken as a baseline that was subtracted from both the wildtype and the mutant conditions. The pmol/μg/min in the mutant was then normalized to the wildtype from each experiment, which was arbitrarily taken as 100%.

### Live cell confocal microscopy and image acquisition

Live cell confocal microscopy was performed using an inverted Zeiss laser scanning microscope (Model 510) with a 63 × 1.4 NA oil immersion lens, 2–2.5 × zoom, and multi-track excitation. HEK 293 T cells were plated on poly-D-lysine-coated, glass-bottom imaging dishes at the density of 1–2 × 10^5^ cells and cotransfected with 2 μg of the wildtype or the mutant GAT-1 plasmids and 1 μg pECFP-ER with PEI based on our standard lab protocol. Cells were examined with excitation at 458 nm for ECFP, 514 nm for EYFP. All images were single confocal sections averaged from 8 times to reduce noise, except when otherwise specified. The images were acquired using a LSM 510 invert confocal microscope with 63X objective.

### Protein structural modeling and machine learning tools

We simulated the impact of the mutation on the transport protein with multiple machine learning tools. Tertiary structures of both the wildtype and P361T mutated GAT-1 protein were predicted by I-TASSER [52] and analyzed by MAESTRO web [29]. Details in structural differences between the wildtype and the mutant GAT-1 were illustrated using the modelled structure by DynaMut [39]. Analysis of self-aggregation or co-aggregation was conducted using PASTA 2.0 [43].

### Data analysis

Numerical data were expressed as mean ± SEM. Proteins were quantified by Odyssey software and data were normalized to loading controls and then to wildtype transporter proteins, which was arbitrarily taken as 1 in each experiment. The radioactivity of GABA uptake was measured in a liquid scintillator with QuantaSmart. The flux of GABA (pmol/μg/min) in the WT GAT-1 samples was arbitrarily taken as 100% each experiment. The fluorescence intensities from confocal microscopy experiments were determined using MetaMorph imaging software and the measurements were carried out in ImageJ as modified from previous description [23, 27, 46]. For statistical significance, we used one-way analysis of variance (ANOVA) with Newman-Keuls test or Student’s unpaired *t*-test. In some cases, one sample *t*-test was performed (GraphPad Prism, La Jolla, CA), and statistical significance was taken as *p* < 0.05.

## Results

### Mutation analysis identified P361T variation in SLC6A1 and the residue is conserved across species

**Fig. 1:**
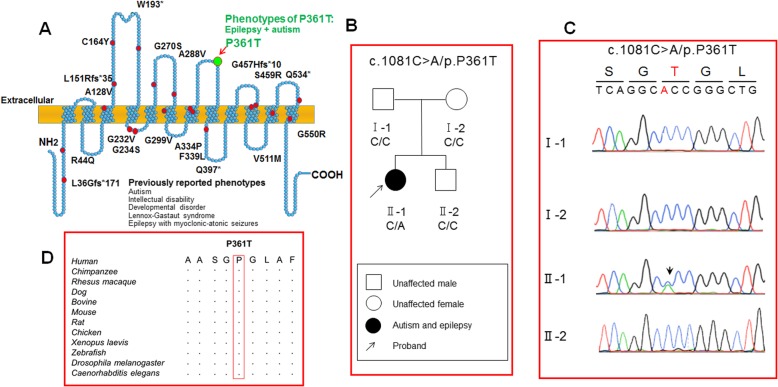
GABA transporter 1 (GAT-1) protein topology, mutations and identification of a novel *SLC6A1* missense mutation GAT1(P361T). **a.** Schematic representation of GAT-1 protein topology and locations of GAT-1 variants previously identified in patients associated with a spectrum of epilepsy syndromes. It is predicted that GAT-1 contains 12 transmembrane domains. P361 is located at the extracellular loop between the 7th and 8th transmembrane helices of the GAT-1 protein. The positions of variants are based on the published LeuT crystal structure. **b** Pedigree and the genotype. A missense mutation was only found in the proband but not in the rest of the family members. **c** Chromatogram of PCR-Sanger sequencing. DNA sequences of the proband and the immediate family members were shown. Arrow indicated a C-to-A transversion. **d** Amino acid sequence homology shows that proline (P) at residue 361 is highly conserved in *SCL6A1* in humans (Accession NO.NP_003033.3) and across species as shown in boxed region

Multiple mutations have been identified in the GAT-1 protein (Fig. 1a) [10, 19, 32]. These mutations are scattered throughout the transporter protein peptide. Protein sequence alignment indicates that P361T in GAT-1 occurs at a conserved residue located at the extracellular loop between the 7th and 8th transmembrane helices. The mutation was identified by whole exome sequencing in the proband but not in her unaffected parents and brother. Initially, all rare and potentially damaging variants were obtained through population and functional impact-based filtration. Next, de novo variants, homozygous and compound heterozygous genotypes in the proband were screened. Finally, according to the clinical concordance evaluation between the previously reported phenotypes of the mutated genes and the phenotypic characteristic of the patient, a de novo novel heterozygous missense variation (Ref Seq accession number NM_001348250: c.1081C > A/p. Pro361Thr) was identified in *SLC6A1* (coding for GAT-1). The proband was confirmed to harbor the variant by Sanger resequencing, but her unaffected family members did not (Fig. 1b and c). Further, this variation was absent in the general population of the 1000 Genomes Project, ExAC, ExAC-EAS, GnomAD, and our inner 296 normal controls. It was predicted to be “damaging” by SIFT (score = 0.0), “probably damaging” by Poly-Phen-2 (score = 1.0), and “disease causing” by Mutation Taster (score = 1). The pathogenicity of the novel *SLC6A1* variant was assessed as likely pathogenic by American College of Medical Genetics and Genomics (ACMG) scoring. This *SLC6A1* variant was the only de novo variant detected via whole exome sequencing. Additionally, four compound heterozygous variants in the *BTN1A1, FLNC, C2CD3,* and *RYR1* genes were detected in the proband, but the clinical phenotypes of these four genes were not in concordance with the clinical features of the proband, suggesting none of these recessive variants were disease-causing variants. No homozygous variants were detected in the proband. We have aligned the encoded GAT-1 sequence and identified the proline residue is conserved across species.

### Protein structural modeling suggests that P361T mutation in GAT-1 protein destabilizes the transporter protein conformation

**Fig. 2:**
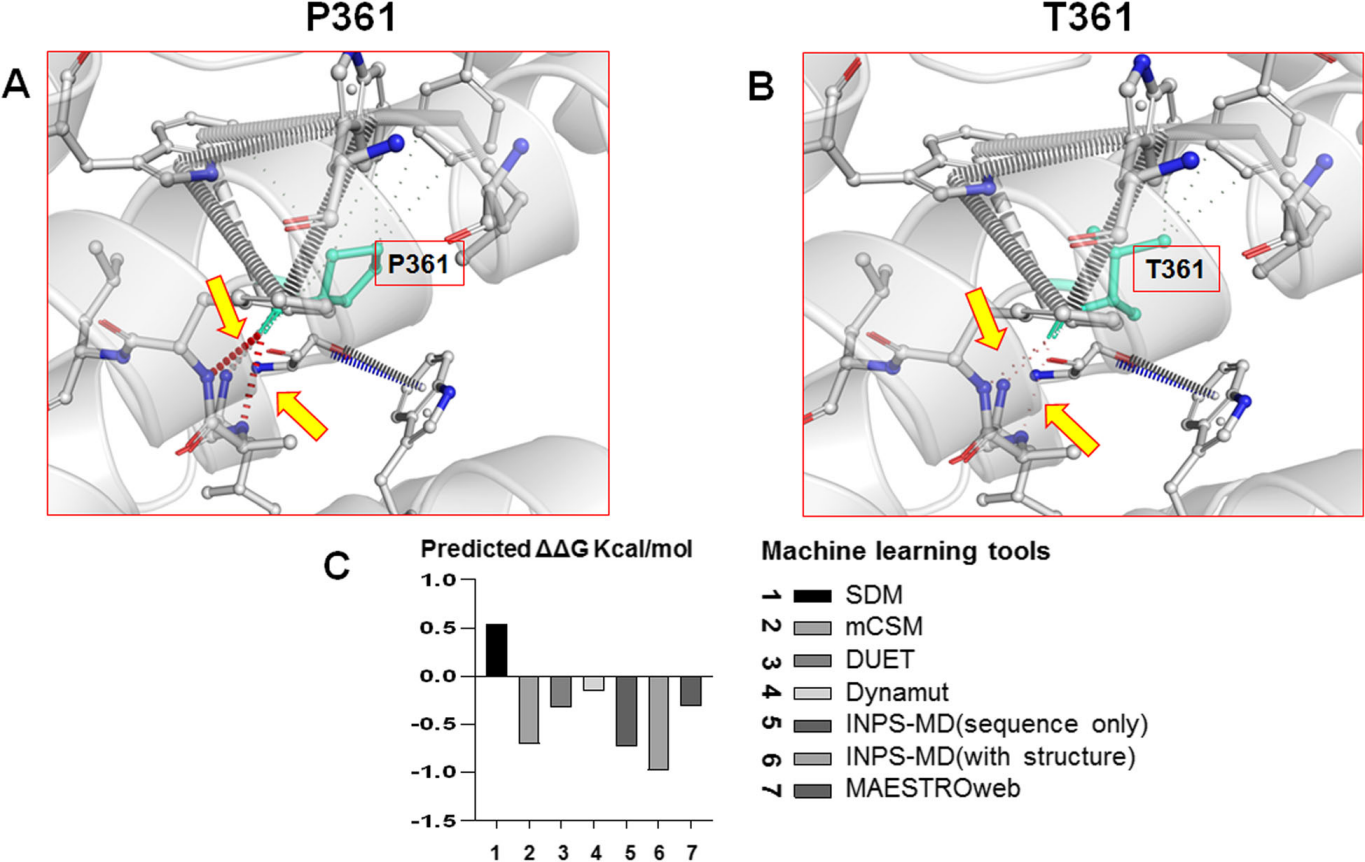
Modeling of the mutant GAT-1 protein with machine learning tools. **a-b**. Tertiary structures of both the wildtype (**a**) and P361T mutant (**b**) GAT-1 protein are predicted by I-TASSER and DynaMut. The proline at residue 361 is mutated to threonine, both highlighted in light green, alongside with the surrounding residues. The interatomic interactions were predicted by DynaMut, where halogen bonds are depicted in blue and hydrogen bonds are colored in red. The P361T mutation results in the loss of two hydrogen bonds, those between residues 361 and 365 (yellow arrow with red border) and between 361 and 364 (yellow arrow with blue border). This supports the result in Table 1 that this mutation destabilized the global conformation of the GAT-1 protein. **c**. Machine learning tools predicted ΔΔG (Kcal/mol) of the mutant GAT-1 protein. Bars in the positive direction are predicted as stabilizing while bars in the negative direction are predicted as destabilizing

We then predicted the impact of the mutation on the transporter protein stability via several machine learning tools. Homology modelling of the P361T mutation in GAT-1 protein as shown in Fig. 2 was conducted using I-TASSER [52] with homology template PDB ID 4 m48. Residue P361T is colored red, where proline is mutated to threonine, which may trigger several conformational changes on GAT-1. Located at the extracellular loop between transmembrane domains, residue 361 is at the turn of two helices exposed on the surface of the protein’s tertiary structure. Similar to the amino acid change from glycine to serine that we reported before on residue 234 (G234S) associated with LGS [8], the additional hydroxyl in threonine increased side chain polarity in comparison to the nonpolar side chain pyrrolidine in the wildtype proline. These polarity changes disturb the equilibrium of the transmembrane protein conformation, resulting in protein structure destabilization. Another observation resulting from the mutation is the breakage of hydrogen bonds between residue 361 and its neighboring residues 365 and 364 in the helix (red dash in Fig. 2a and b). This destabilization hypothesis is also supported by predicting the ΔΔG of the mutation using machine learning-based protein structure stability prediction methods SDM [35], mCSM, DUET [36], INPS [2, 40], DynaMut [39] and MAESTROweb [29]. As indicated in Fig. 2c Supplementary Table 1 [29, 36, 37, 39, 40], nearly all the tools (six out of seven) predicted the P361T mutation destabilized the GAT-1 protein (Supplementary Table 1). Details in structural differences between the wildtype proline and mutated threonine were modelled by DynaMut interatomic interaction predictions. In addition, PASTA 2.0 [43] did not suggest any protein self-aggregation or co-aggregation from the perspective of energy changes.

### Clinical phenotypes of autism and epilepsy

The proband, a 6-year-old girl was diagnosed as autism and epilepsy at 3.5 years old. She was born to non-consanguineous healthy parents, termed and delivered naturally. There was no history of ASD, epilepsy, development disorders, or other neurological disorders in her family members or other relatives. The patient had developmental delay in gross and fine motor skills and speech at 6 months. Two month later, she had repetitive patterns of behavior, such as playing with hair, wringing hands, tapping the desk, and grinding her teeth. Diminished social interactions occurred, such as poor eye contact and no attempt to interact with any member of family, even her mother. She only engaged in solitary play, was unable to inform her parents about her needs, and was unconcerned about any change in the immediate environment.

**Fig. 3:**
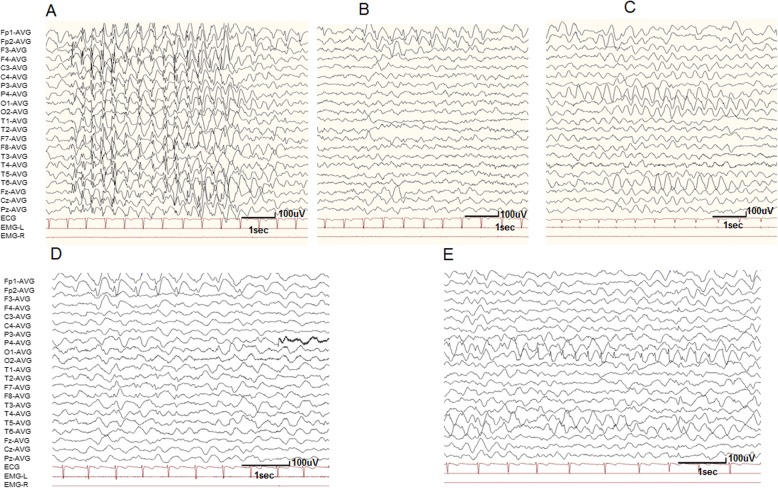
Electroencephalogram (EEG) of a 6-year-old girl carrying GAT-1(P361T) mutation. Interictal video EEG recordings showed 2.5–3.0 Hz generalized spike and slow waves (**a**), 2.0–3.0 Hz spike and slow waves in the bilateral prefrontal lobes (**b**) and 2.0–3.0 Hz slow waves predominantly in the bilateral occipital area (**c**) during both wakefulness and sleep when the patient was 3.5 years old. Interictal video EEG recordings demonstrated 2.0–3.0 Hz spike and slow waves in the bilateral prefrontal lobes (**d**), and 2.0–3.0 Hz spike and slow waves predominantly in the bilateral occipital and posterior-temporal area (**e**) during both wakefulness and sleep when the patient was 6 years old

At 41.8 months of age, she was first evaluated in a psychology clinic with ADI-R and ADOS, receiving scores in each domain of the ADI-R and ADOS much higher than that the cut-off values of ASD (Tables 1, 2 and 3) and was consequently diagnosed with ASD (Tables 1, 2 and 3). The assessment results of CPEP-3 and Gesell indicated that she had regression in behaviors, developmental skills, and neuropsychological development (Tables 4 and 5). Additionally, the patient had her first seizure at 2 years old without obvious predisposing factors. It was a series of repetitive absence seizure attacks with transient loss of consciousness for 3–5 s, accompanied by an atonic seizure with head drooping to one side and occasionally to the ground. Subsequently, similar seizures occurred more than 10 times per day. Her brain MRI was normal. Interictally EEG recordings showed 2.5–3.0 Hz generalized spike and slow waves (Fig. 3a), spike and slow waves in the bilateral prefrontal lobes, and slow waves (2.0–3.0 Hz) predominantly in the bilateral occipital area during both wakefulness and sleep (Fig. 3a-c). A diagnosis of generalized epilepsy was considered. The patient had not received AED therapy before 3.5 years old. She was initially treated with valproate (VPA) with a dose of 20 mg.kg^-l^d^− 1^; the seizure frequency significantly reduced but the odontoprisis was still observed. She was then treated with levetiracetam (LEV) with a dose of 28.57 mg.kg^-l^d^− 1^. The seizures and odontoprisis disappeared, but she became irritable with frequent screaming. Finally, lamotrigine (LTG) was used as a substitute for LEV with a dose of 4.16 mg.kg^-l^d^− 1^, and her condition was more stabilized than before. A recent EEG recording demonstrated the generalized epileptic discharge disappeared, but the focal EEG abnormalities did not show significant improvement (Fig. 3d, e).

**Table 1:** Clinical features of the patient with P361T variants in *SLC6A1*

Patient ID	
Variant	c.1081C > A (NM_001348250)
Protein change	p.P361T
Origin	De novo
Sex	Female
Current age	6 years
Age at seizure onset	2 years
Seizure type at onset	Absence and atonic
Seizure frequency at onset	10 times per day
Interictal EEG	GSW, focal SW
Seizure outcome	Seizure free
Duration before seizure free	3 years
MRI findings	Normal
ADI-R RSI	24
ADI-R COM	14
ADI-R RRB	4
ADI-R abnormality of development at or before 36 months	5
ADOS SC	6
ADOS SA	14
Intellectual disability	Severe ID
Language	Speech delay
Behaviors and developmental skills	Dysfunctional
Neuropsychological development	Severer or profound retardation
Diagnosis	Autism and generalized epilepsy
SIFT (score)	Damaging (0.0)
Polyphen2 (score)	Probably damaging (1.0)
Mutation Taster (score)	Disease causing (1)
Frequency in gemomeAD_exome	–
Frequency in ExAC	–
Frequency in ExAC (East Asian)	–
Frequency in 1000 genomes	–

*ADI-R* Autism Diagnostic Interview-Revised, *ADOS* Autism Diagnostic Observation Schedule; *COM* qualitative abnormalities in communication, *EEG* electroencephalography, *GSW*: generalized spike and slow wave, *ID intellectual disability, MRI* magnetic resonance imaging, *RSI* qualitative abnormalities in reciprocal social interaction, *RRB* restricted and repetitive behavior, *SA* social affect, *SC* social communication, *SW* spike and wave complex

**Table 2:** Autism Diagnostic Interview-Revised (ADI-R) of the patient at the age of 41.8 months

Domain	Score
**Qualitative Abnormalities in Reciprocal Social Interaction (RSD)**	
Failure to use nonverbal communication to regulate social interaction (A1)	6
Failure to develop peer relationships (A2)	4
Lack of shared enjoyment (A3)	6
Lack of socioemotional reciprocity (A4)	8
Subtotal (Cut-off)	24 (10)
**Qualitative Impairments in Communication and Language (COM)**	
Lack of, or delay in, spoken language and failure to compensate through gesture (B1)	8
Lack of varied spontaneous make-believe or social imitative play (B4)	6
Subtotal (Cut-off)	14 (7)
**Restricted, Repetitive, and Stereotyped Behaviors and interests (RRB)**	
Encompassing preoccupations or circumscribed pattern of interest (C1)	0
Apparently Compulsive Adherence to Nonfunctional Routines or Rituals (C2)	0
Stereotyped and repetitive motor mannerisms (C3)	2
Preoccupation with part of objects or nonfunctional elements of material (C4)	2
Subtotal (Cut-off)	4 (3)
**Abnormality of development evident at or before 36 months (Cutoff)**	5 (1)
**Total (**Cut-off**)**	47 (21)

*COM* qualitative abnormalities in communication, *RRB* restricted and repetitive Behavior; *RSI* qualitative abnormalities in reciprocal social interaction

**Table 3:** Autism Diagnostic Observation Schedule (ADOS) of the patient at the age of 41.8 months

Domain	Score
**Language and Communication (SC)**	
Frequency of spontaneous vocalization directed to others (A2)	2
Stereotyped/Idiosyncratic use of words or phrases (A5)	0
Use of another’s body (A6)	0
Pointing (A7)	2
Gestures (A8)	2
Subtotal (AUT, AS)	6 (4, 2)
**Reciprocal Social Interaction (SA)**	
Unusual eye contact (B1)	2
Facial expression directed to others (B3)	2
Share enjoyment in interaction (B5)	2
Showing (B9)	2
Spontaneous initiation of joint attention (B10)	2
Response to joint attention (B11)	2
Quality of social overtures (B12)	2
Subtotal (AUT, AS)	14 (7, 4)
Total of SA and SC (AUT, AS)	20 (12, 7)
**Play**	
Functional play with objects (C1)	2
Imagination/Creativity (C2)	2
Subtotal	4
**Stereotyped Behaviors and Restricted Interests (RRB)**	
Unusual sensory interest in play material/person (D1)	1
Hand and finger and other complex mannerisms (D2)	2
Unusually repetitive interests or stereotyped behaviors (D4)	0
Subtotal	3

*AS* autism spectrum cut-off, *AUT* autism cut-off; *SA* Social Affect, *SC* Social Communication, *RRB* Restricted and Repetitive Behavior

**Table 4:** Chinese Psychoeducational Profile-Third Edition (CPEP-3) of the patient at the age of 41.8 months

CPEP-3 subsets	Raw score	Development ages (months)	Percentiles	Developmental /adaptive levels
**Performance Test**				
CVP	0	< 12	< 2	Severe
EL	0	< 12	2	Severe
RL	0	< 12	2	Severe
FM	1	< 12	< 2	Severe
GM	3	< 12	< 2	Severe
VMI	0	< 12	2	Severe
AE	7	–	6	Severe
SR	1	–	< 2	Severe
CMB	9	–	4	Severe
CVB	0	–	2	Severe
**Caregiver Report**				
PB	0	–	< 2	Severe
PSC	0	< 12	< 2	Severe
AB	4	–	< 2	Severe
**Composites**	**Standard score**	**Development ages (months)**	**Percentiles**	**Developmental** **/adaptive levels**
C (CVP + EL + RL)	14	6.0	6	Severe
M (FM + GM + VMI)	4	6.0	1	Severe
MB (AE + SR+ CMB + CVB)	14	–	2	Severe

*AB* adaptive behavior, *AE* affective expression, *CMB* characteristic motor behaviors, *CVB* characteristic verbal behaviors, *CVP* cognitive verbal/preverbal, *EL* expressive language, *FM* fine motor, *GM* gross motor, *PB* problem behaviors, *PSC* personal self-care, *RL* receptive language, *SR* social reciprocity, *VMI* visual-motor imitation

**Table 5:** Gesell developmental schedule of the patient at the age of 41.8 months

Subfields	DA (month)	DQ	Assessment
Gross motor	14.23	34	Severe
Fine motor	7.47	18	Profound
Adaptive behavior	4.2	10	Profound
Language	5.83	14	Profound
Personal-social ability	5.37	13	Profound

*DA* Development Age, *DQ* Development Quotient

### GAT-1(P361T) had reduced total protein in both non-neuronal cells and mouse cortical neurons

**Fig. 4:**
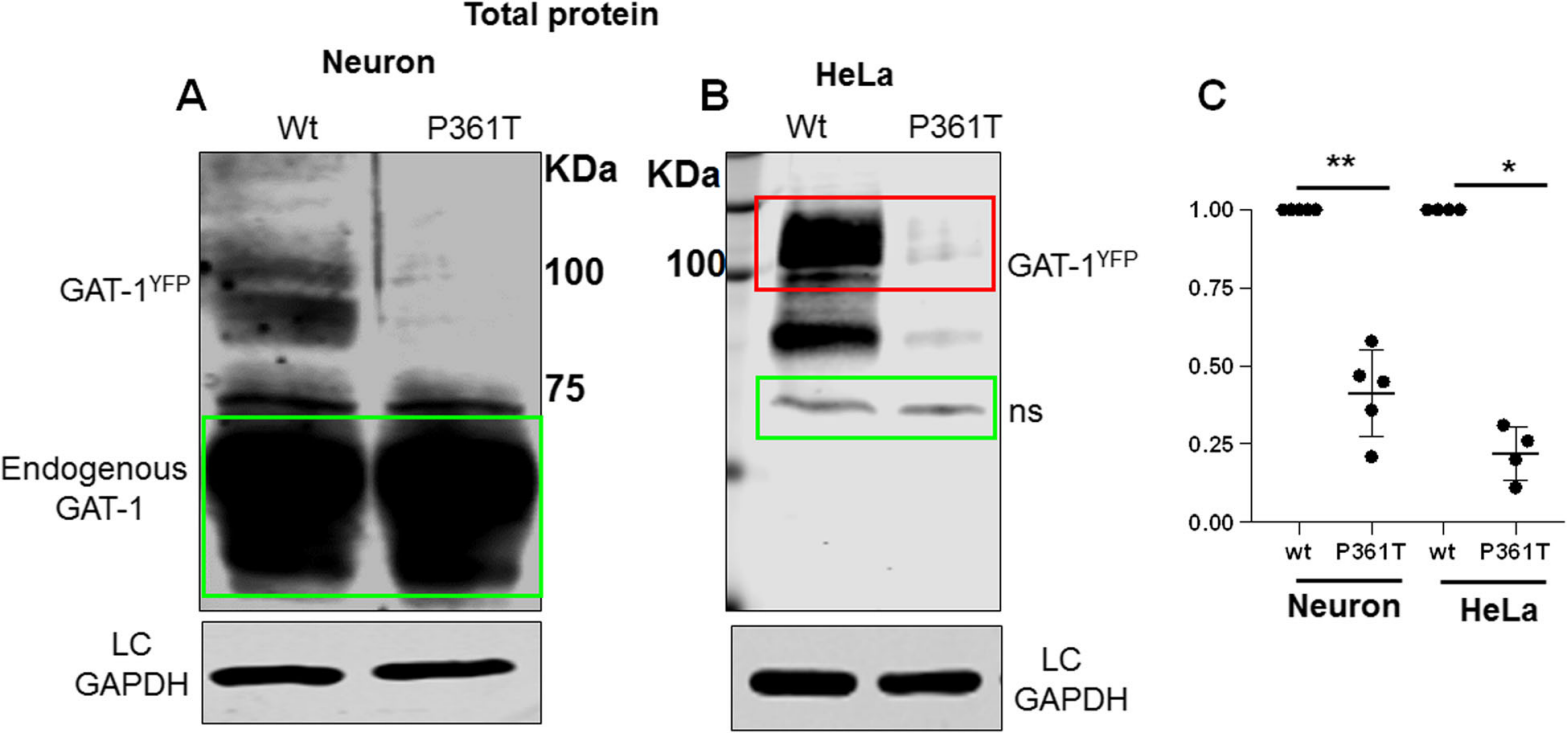
The expression of the mutant of GAT-1(P361T) protein was reduced in non-neuronal cells and neurons. **a-b.** Mouse cortical neurons were transfected with the wildtype or the mutant GAT-1(P361T) cDNAs at day 7 in culture. The total lysates were harvested from mouse cortical neurons expressing the wildtype GAT-1^YFP^ (wt) or mutant GAT-1(P361T)^YFP^ transporters after 8 days of transfection (**a**). HeLa cells were transfected with the wildtype GAT-1^YFP^ (wt) or mutant GAT-1(P361T)^YFP^ transporters for 48 h (**b**). The total lysates were then analyzed by SDS-PAGE. Membranes were immunoblotted with rabbit anti-GAT-1 for both neuronal and HeLa cell lysates (1:200). In neurons, the protein band of endogenous GAT-1, at 67 KDa, was intense. The main protein bands run at 108 KDa in both the wildtype and the mutant conditions, representing the YFP-tagged GAT-1. **c**. The total protein integrated density values (IDVs) were measured. The abundance of the mutant GAT-1(P361T) transporter was normalized to the wildtype condition. In **c**, the total protein abundance was measured by adding up all the bands between 90 and 110 KDa. The total protein IDVs of either the wildtype or the mutant was normalized to its loading control. The abundance of the mutant transporter was then normalized to the wildtype. (*p < 0.05 vs wt in HeLa; **p < 0.01 vs. wt in Neuron, *n* = 4–5 different transfections)

Altered protein stability and enhanced protein degradation are common phenomena caused by mutations in various genes. This has been demonstrated in multiple GABA_A_ receptor mutations across multiple subunits [26]. We first determined the total expression of the mutant GAT-1(P361T) by transfecting mouse cortical neurons with YFP-tagged wildtype or mutant GAT-1 cDNAs (Fig. 4a) for 8 days. We also transfected the YFP-tagged wildtype or mutant GAT-1 cDNAs in HeLa cells for 48 h. In both neurons and HeLa cells, the wildtype GAT-1^YFP^ or the mutant GAT-1(P361T)^YFP^ mainly migrated at 108 KDa, which is predicted for YFP-tagged GAT-1 and is consistent with previous findings [5, 7]. When immunoblotted with anti-GAT-1 antibody, a strong band was detected at 67 KDa. This is the endogenous GAT-1, which was not changed in neurons transfected with either the wildtype or the mutant GAT-1 cDNAs (data not shown). This may suggest there is no dominant negative effect of the mutant GAT-1(P361T) in neurons (Fig. 4). Compared to the wildtype, the GAT-1(P361T) had reduced total protein expression (wt = 1, P361T = 0.22 ± 0.043) in mouse cortical neurons (Fig. 4a) and in HeLa cells (wt = 1, P361T = 0.41 ± 0.062) (Fig. 4b-c). This suggests a similar reduction of the total protein level in the mutant GAT-1 in neurons and non-neuronal cells.

### GAT-1(P361T) mutant protein was retained inside the endoplasmic reticulum

**Fig. 5:**
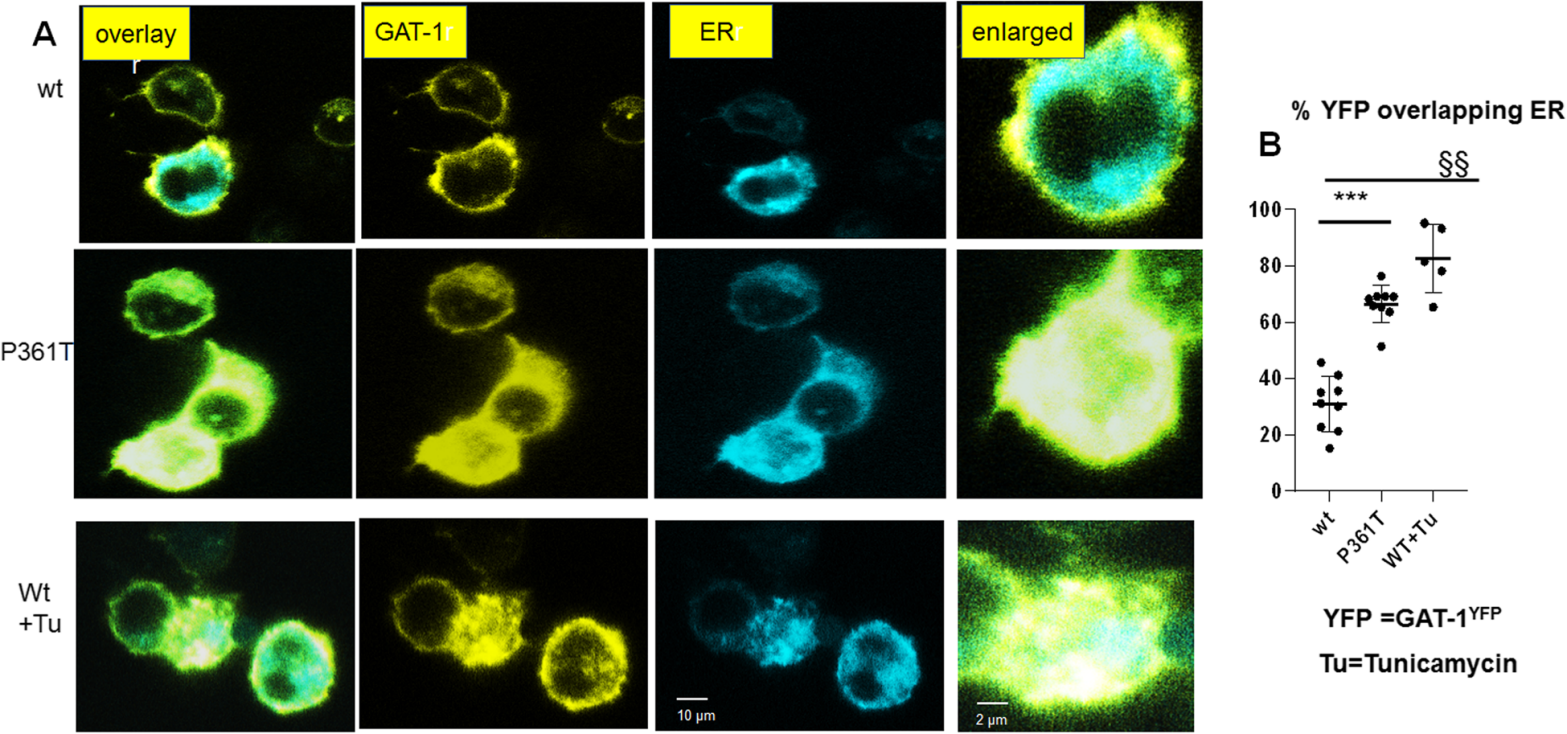
There was reduced YFP fluorescence in cells expressing the mutant GAT-1(P361T) transporters, which were retained inside the endoplasmic reticulum. **a** HEK293T cells were transfected with wildtype GAT-1^YFP^ or the mutant GAT-1(P361T)^YFP^ with the pECFP-ER marker (ER^CFP^) at 2:1 ratio (2 μg:1 μg cDNAs) for 48 h. Live cells were examined under a confocal microscopy with excitation at 458 nm for CFP, 514 nm for YFP. All images were single confocal sections averaged from 8 times to reduce noise, except when otherwise specified. **b** The GAT-1^YFP^ fluorescence overlapping with ER^CFP^ fluorescence was quantified by Metamorph with colocalization percentage. (***p < 0.001 P361T vs. wt, §§ *p* < 0.01 wt + Tunicamycin vs wt untreated, *n* = 5–9 representative fields from different transfections)

We have previously identified that mutant GABA_A_ receptor subunits are more likely to be retained inside the endoplasmic reticulum (ER) due to misfolding and glycosylation arrest [25, 26]. Those ER retention-prone mutant proteins can have either a higher or lower proportion of the total protein level compared with its wildtype counterpart [25, 26]. To evaluate the subcellular localization of GAT-1(P361T), we determined the intracellular localization of the mutant GAT-1 (P361T) protein by coexpressing GAT-1^YFP^ or GAT-1(P361T)^YFP^ with an ER marker, ER^CFP^ [25]. When compared to wildtype, the mutant GAT-1(P361T) had a stronger presence intracellularly, colocalizing with the ER marker (Fig. 5a). The protein expression pattern was very similar to that of the wildtype GAT-1 protein treated with ER stress inducer tunicamycin (10μg/ml for 16 h). The percent fluorescence signal of GAT-1 overlapping with ER marker was higher in the mutant GAT-1(P361T) compared to wildtype (30.90 ± 3.26 vs 66.48 ± 2.23) (Fig. 5b). The percent fluorescence signal of GAT-1(P361T) overlapping with ER marker was similar to the wildtype treated with tunicamycin (66.48 ± 2.23 vs 82.24 ± 5.428) (Fig. 5b). The data indicates that the mutant protein was more likely to be retained inside the ER despite the reduced total amount of the mutant GAT-1(P361T) protein.

### GAT-1(P361T) had compromised GABA reuptake when evaluated with ^3^H-labeled radioactive GABA transport assay

**Fig. 6:**
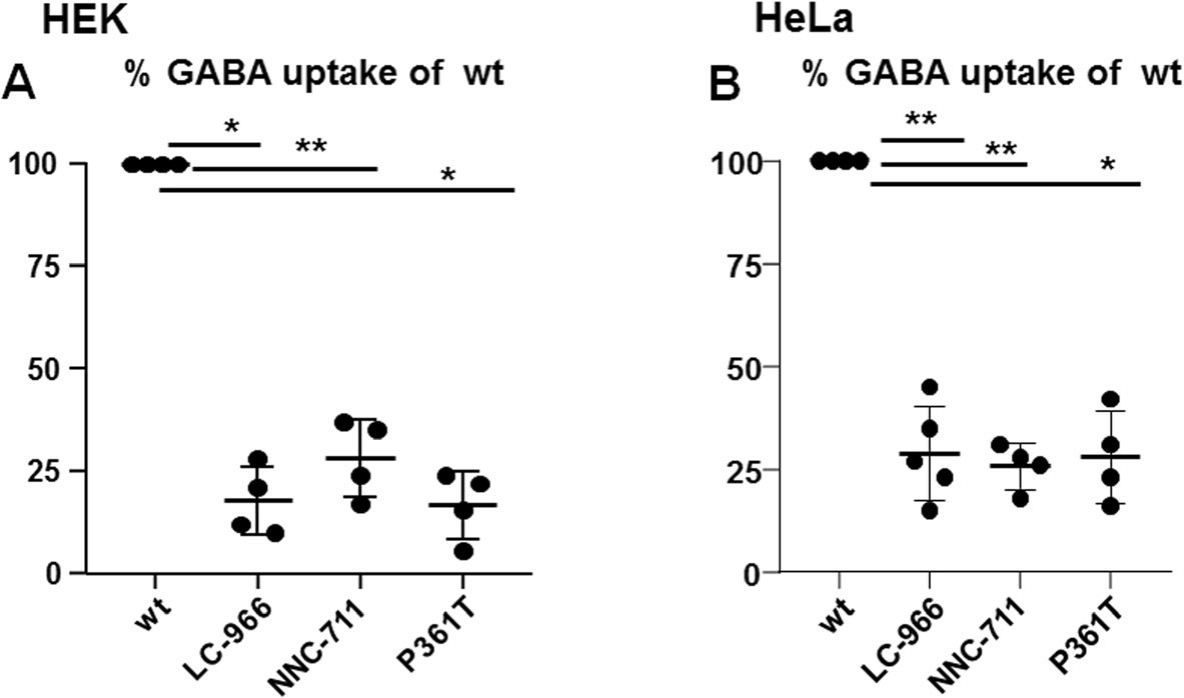
Impaired GABA uptake of the mutant GAT-1(P361T) transporters. (**A**) HEK293T cells were transfected with wildtype GAT-1^YFP^ (wt), or the mutant GAT-1(P361T)^YFP^ cDNAs (1 μg/35mm^2^) for 48 h. The GABA uptake assay was carried out with ^3^H radioactive labeling in HEK 293 T cells. GABA flux was measured after 30 min transport at room temperature. The influx of GABA, expressed in pmol/μg protein/min, was averaged from duplicates for each condition and for each transfection. The average counting was taken as *n* = 1. The untransfected condition was taken as baseline flux, which was subtracted from both the wild-type and the mutant conditions. The pmol/μg protein/min in the mutant was then normalized to the wildtype from each experiment, which was arbitrarily set as 100%. (**p < 0.01 vs. wt, n = 4–5 different transfections)

The reduced total expression of the mutant GAT1(P361T) could consequently impair the overall function of GAT-1 due to the reduced number of functional transporters. We then determined the function of the wildtype and the mutant GAT-1 (P361T) in HEK cells by ^3^H GABA uptake assay. The flux was conducted in a preincubation solution containing 1μci/ml and 10 μM cold GABA at room temperature for 15 min. The counts per minute (CPM) were converted to pmol/μg/min by normalizing to the standard CPM, protein concentration, and time for flux. The measurements in the mutant transporter were then normalized to the wildtype which was taken as 100%. Compared with the wildtype, the GAT-1(P361T) had reduced ^3^H GABA uptake in both HEK293T (wt = 100% vs 16.83 ± 4.1) (Fig. 6a) and HeLa (wt = 100% vs 28.0 ± 5.58) cells (Fig. 6b). The GAT-1 (P361T) transport activity was similar to the activity of wildtype GAT-1 treated with GAT-1 inhibitors Cl-966 (100 μM) and NNC-711 (70 μM) for 30 min in both HEK293T and HeLa cells. This indicated that the P361T mutation reduced the transporter activity to a similar level as that of the cells treated with the GAT-1 inhibitors Cl-966 or NNC-711.

## Discussion

### Mutations in *SLC6A1* are associated with a wide spectrum of clinical phenotypes including autism and epilepsy

It has been previously reported that MAE and ID are the two prominent phenotypes for *SLC6A1* mutations [9, 19]. More recently, studies on clinical manifestations associated with *SLC6A1* variants indicate that variants in *SLC6A1* can give rise to a wide spectrum of epilepsy syndromes, ranging from focal epilepsy to generalized epilepsy as well as learning disorders and intellectual disability with or without epilepsy [2]. Our study supports the hypothesis that mutations in *SLC6A1* could give rise to a wide spectrum of epilepsy phenotypes. We have previously reported that a *SLC6A1* mutation is associated with LGS. Here we are the first to report that a *SLC6A1* missense mutation causes ASD plus epilepsy. Expanding the phenotype spectrum associated with *SLC6A1* mutations and further supporting our previous hypothesis [19] that mutations in *SLC6A1* are associated with a wide spectrum of phenotypes. However, the mechanisms underlying the phenotypic heterogeneity merits further elucidation.

### *SLC6A1* mutation mediated phenotypes suggest a role of GAT-1 in early brain development

It has been reported that the head circumference is increased in autistic toddlers [11, 12]. The genetic risk factors for autism range from rare point mutations in genes encoding numerous synaptic proteins (such as contactin-associated protein-like 2, CNTNAP2; SH3 and multiple ankyrin repeat domains 3, SHANK3; and neuroligin 3, NLGN3), to gains or losses of DNA segments, termed copy number variation (for example, 16p11.2 and 15q11-q13), and to gross chromosomal rearrangements that are estimated to occur in about 7% of autism cases [1, 34]. It has been identified that ASD genes as a group are preferentially expressed in late mid-fetal prefrontal cortex and have concentrated expression in layer V/VI cortical projection neurons [47]. Collectively, studies from both rare mutations and common variants highlight the relevance of early fetal brain development in the pathophysiology of ASD (). Although the developmental profile of GAT-1 is unclear, it is likely that GAT-1 plays an important role in early brain development via affecting GABA signaling.

### Impaired GABAergic signaling, a converging pathway in autism and epilepsy

GABA is a critical neurotrophic signal in early brain development [44, 45]. GABA modulates neuronal arbor elaboration and differentiation. In chick cortical and retinal cells, treatment with GABA increased the length and branching of the neurites and augmented the density of synapses. In mammalian neurons, GABA_A_ receptor antagonists reduced the dendritic outgrowth of cultured rat hippocampal neurons. In subsequent studies in diverse brain structures, including cerebellar granule cells, cortical plate and subplate interneurons, spinal cord cells, and raphe nuclei 5-hydroxytryptamine (serotonin)-producing neurons, the trophic action of GABA showed similar results. The trophic effects of GABA have been reproduced by agents acting on GABA synthesis, receptor activation or blockade, intracellular Cl^_^ homeostasis, or L-type Ca^2+^ channels. Similarly, conversion of GABA-induced excitation/depolarization into inhibition/hyperpolarization in newborn neurons leads to significant defects in their synapse formation and dendritic development in vivo [16]. It has been demonstrated that GABA_A_ receptor activation impacts neurite growth in various systems [6, 31, 42], validating the critical role of GABA signaling in brain development; however, the expression profile of GAT-1 in early brains and how impaired GAT-1 function affects early progenitor cells are unknown.

### Mutations in *SLC6A1* cause clinical phenotypes similar to mutations in *GABRB3,* suggesting overlapping pathophysiology underlying mutations in *SLC6A1* and *GABRB3*

Both GABA_A_ receptors and GAT-1 are key components of the GABAergic signaling pathway. It is not surprising that mutations in genes encoding both GABA_A_ receptor subunits and GAT-1 are associated with the same clinical epilepsy phenotype. It is plausible that GABA_A_ receptors and GABA transporters like GAT-1 work in concert to ensure an appropriate level of GABAergic neurotransmission as well as proper neurotrophic signaling during the progenitor cell stage. It has been demonstrated that *GABRB3* affects cell proliferation and differentiation [3] at the stem cell stage. It is possible that mutations affecting either GABA_A_ receptors or GABA transporters such as GAT-1 can impair GABAergic signaling and give rise to a similar clinical presentation. However, it merits further study to elucidate the similarity and difference of mutations in both genes from functional evaluations to clinical phenotypes, especially regarding the impact on early progenitor cell differentiation, spatial localization, and neuronal maturation.

### Mutant GAT-1(P361T) protein had reduced protein stability and reduced total protein expression

Our findings from protein structure simulation by machine learning, as well as appropriate modeling with various tools, indicate the P361T substitution results in the breakage of hydrogen bonds between residue 361 and its neighboring residues 365 and 364 in the helix, as indicated by structural simulation. Collectively, simulation data indicate that the mutation destabilizes the protein conformation. Our biochemical assay has demonstrated that the GAT-1(P361T) mutation reduced the total protein expression in both heterologous cells and neurons, further validating the hypothesis of reduced GAT-1 protein stability. Based on the wildtype and mutant protein expression patterns, it is likely that the mutation only caused a partial loss of function without clear dominant negative effects (data not shown).

### Mutant GAT-1(P361T) transporter was mislocalized with increased ER retention

We previously demonstrated that mutant GABA_A_ receptor subunits were retained inside the ER and were removed from the cells by ER-associated degradation, and that this is a major pathogenicity for GABA_A_ receptor subunit gene mutations. Because GAT-1 is a transmembrane protein, it is likely that at least some mutations in GAT-1 cause protein instability and impair trafficking. We evaluated the subcellular colocalization of GAT-1(P361T) with an ER marker, and also evaluated the colocalization of wildtype GAT-1 with the ER marker after treatment with an ER stress inducer, tunicamycin (10 μg/ml). The GAT-1(P361T) expression profile was highly colocalized with the ER marker. The findings were similar to the expression pattern of the wildtype GAT-1 treated with tunicamycin or with Brefeldin A (which blocks protein transport from the ER to the Golgi apparatus) (Data not shown). Our data indicate that the mutant GAT-1(P361T) transporter is subject to similar intracellular protein processing as many mutant GABA_A_ receptor subunits, due to a conserved protein quality control machinery inside cells [25, 26]. The steady state level of the ER retained mutant protein could be higher or lower than its wildtype counterparts, depending on the intrinsic properties of the mutant protein that effect degradation rate of the mutant protein (Kang et al., 2009 c[24];). GAT-1(P361T) had reduced total protein expression in both neurons and non-neuronal cells, indicating reduced protein stability and enhanced disposal of the mutant protein, and most of the synthesized mutant transporters resided inside the ER. This finding is novel for *SLC6A1* mutations but is consistent with our previous studies on multiple GABA_A_ receptor subunit mutations associated with genetic epilepsy syndromes [25, 27].

### GAT-1(P361T) compromises the function of the transporter on GABA uptake

GABA uptake assay is a gold standard to evaluate the function of GABA transporters. Our data indicate that GAT-1(P361T) substantially reduced GABA reuptake to the level of cells expressing the wildtype GAT-1 treated with GAT-1 inhibitors Cl-966 and NNC-711. Biochemical studies and confocal microscopy analysis indicate the total amount of mutant protein was substantially reduced and the remaining protein was likely retained inside ER; consequently, there would be far fewer transporters at the cell surface and synapses to conduct GABA transportation. This indicates GAT-1(P361T) is a loss-of-function mutation and could explain the associated disease phenotype in the patient carrying the mutation.

### The implications of the impact of mutant GAT-1 on early brain development and neurodevelopmental disorders

It is likely that mutations in GAT-1 cause dysregulated cell proliferation and differentiation at the early progenitor cell stage, which is very similar to the impact of GABA_A_ receptor mutations. Future studies with human patient-derived pluripotent stem cells and animal models have potential to elucidate the pathological basis for these effects. Additionally, GAT-1 is a major target for seizure treatment. Tiagabine (TGB) is an inhibitor of GAT-1 and is widely used in focal epilepsy. How can loss-of-function mutations in GAT-1 cause epilepsy while inhibiting GAT-1 function paradoxically treats epilepsy? How does the malfunctioning GAT-1 affect tonic and phasic GABA-evoked current? How will seizure suppression with GAT-1 inhibition affect cognition and neurodevelopment? Studies from GAT-1 knockout mice indicate increased tonic current but decreased amplitude of spontaneous miniature inhibitory postsynaptic currents (mIPSCs) [18, 50]. Because the mutant GAT-1(P361T) resulted in reduced GABA uptake, this would likely lead to higher ambient GABA concentration and enhanced tonic inhibition. Future work in mutation knockin mouse models with focus on early brain development, phasic versus tonic inhibition, tailored seizure treatment, and the correlation of seizure treatment and improvement of comorbidities—such as autism and cognition—will be of particular interest. The study has thoroughly characterized the clinic and molecular defects of GAT-1(P361T) mutation. Further study on mutation knockin mouse model would provide critical insights into the change of neurotransmission such as altered tonic and phasic inhibition.

In summary, this study has characterized the clinical features of both epilepsy and ASD phenotypes for SLC6A1 (P361T) mutation and identified the molecular defects with a multidisciplinary approach including ^3^H GABA uptake assay and confocal microscopy. The study indicates the mutation can reduce GAT-1 total expression and GABA uptake. This is likely due to altered GAT-1 protein stability, leading to enhanced GAT-1 protein degradation. Consequently, deficient GAT-1 function may alter neurodevelopment and neurotransmission that manifest as ASD and epilepsy.

## Supplementary information

**Additional file 1 Supplementary Video 1.** Video of 360° rotation animation of GAT-1 protein model (green) with P361T (red) mutation.

**Additional file 2 Supplementary Table 1.** Protein stability prediction on P361T mutation by machine learning methods.

## Data Availability

Any raw data of functional assay can be made available upon request. Any clinical information can be made available upon request subject to approval by the appropriate ethical board.

## **Abbreviations**

ASD: Autistic spectrum disorder
ID: intellectual disability
GAT-1: GABA transporter 1
ER: endoplasmic reticulum
MAE: Myoclonic atonic epilepsy
LGS: Lennox-Gastaut syndrome
AEDs: Antiepileptic drugs
MRI: Magnetic resonance imaging
EEG: Electroencephalography
PEI: Polyethylenimine
