# Supplementary material for: Endoplasmic reticulum retention and degradation of a mutation in SLC6A1 associated with epilepsy and autism
Source: Mol Brain. 2020 May 12;13:76. doi: 10.1186/s13041-020-00612-6 (PMC7218610; doi:10.1186/s13041-020-00612-6)
Supplement: Supplementary file 2 — Additional file 2 Supplementary Table 1. Protein stability prediction on P361T mutation by machine learning methods. [file 13041_2020_612_MOESM2_ESM.docx]

**Supplementary Table 1. Protein stability prediction on P361T mutation by machine learning methods**

| Method Name | Predicted ΔΔG (Kcal/mol) | Outcome | Reference |
| --- | --- | --- | --- |
| SDM | 0.54 | Stabilizing | (Pandurangan, et al., 2017) |
| mCSM | -0.705 | Destabilizing | (Pires et al., 2013) |
| DUET | -0.324 | Destabilizing | (Pires et al., 2014) |
| DynaMut | -0.151 | Destabilizing | (Rodrigues, et al., 2018) |
| INPS-MD(sequence only) | -0.733 | Destabilizing | (Savojardo et al., 2016) |
| INPS-MD(With modelled structrue) | -0.975 | Destabilizing | (Savojardo et al., 2016) |
| MAESTROweb | -0.305 | Destabilizing | (Laimer, et al., 2016) |

Reference:

Laimer, J., Hiebl-Flach, J., Lengauer, D., & Lackner, P. (2016). MAESTROweb: a web server for structure-based protein stability prediction. Bioinformatics, 32(9), 1414-1416.

Pandurangan, Arun Prasad, Bernardo Ochoa-Montaño, David B. Ascher, and Tom L. Blundell. "SDM: a server for predicting effects of mutations on protein stability." Nucleic acids research 45, no. W1 (2017): W229-W235.

Pires, Douglas EV, David B. Ascher, and Tom L. Blundell. "mCSM: predicting the effects of mutations in proteins using graph-based signatures." Bioinformatics 30, no. 3 (2013): 335-342.

Pires, Douglas EV, David B. Ascher, and Tom L. Blundell. "DUET: a server for predicting effects of mutations on protein stability using an integrated computational approach." Nucleic acids research 42, no. W1 (2014): W314-W319.

Rodrigues, Carlos HM, Douglas EV Pires, and David B. Ascher. "DynaMut: predicting the impact of mutations on protein conformation, flexibility and stability." Nucleic acids research (2018).

Savojardo C., Fariselli P., Martelli P.L., Casadio R. "INPS-MD: a web server to predict stability of protein variants from sequence and structure", Bioinformatics (2016).
